# MetalNet2: an enhanced server for predicting metal-binding sites in proteomes

**DOI:** 10.1093/nsr/nwae391

**Published:** 2024-11-05

**Authors:** Feng Zhang, Yao Cheng, Boxin Xue, Yiqin Gao, Yuan Liu, Chu Wang

**Affiliations:** Beijing National Laboratory for Molecular Sciences, College of Chemistry and Molecular Engineering, Peking University, China; Synthetic and Functional Biomolecules Center, Key Laboratory of Bioorganic Chemistry and Molecular Engineering of Ministry of Education, Peking University, China; Beijing National Laboratory for Molecular Sciences, College of Chemistry and Molecular Engineering, Peking University, China; Synthetic and Functional Biomolecules Center, Key Laboratory of Bioorganic Chemistry and Molecular Engineering of Ministry of Education, Peking University, China; Beijing National Laboratory for Molecular Sciences, College of Chemistry and Molecular Engineering, Peking University, China; Beijing National Laboratory for Molecular Sciences, College of Chemistry and Molecular Engineering, Peking University, China; Changping Laboratory, China; Institute of Systems and Physical Biology, Shenzhen Bay Laboratory, China; Beijing National Laboratory for Molecular Sciences, College of Chemistry and Molecular Engineering, Peking University, China; Synthetic and Functional Biomolecules Center, Key Laboratory of Bioorganic Chemistry and Molecular Engineering of Ministry of Education, Peking University, China; Beijing National Laboratory for Molecular Sciences, College of Chemistry and Molecular Engineering, Peking University, China; Synthetic and Functional Biomolecules Center, Key Laboratory of Bioorganic Chemistry and Molecular Engineering of Ministry of Education, Peking University, China; Peking-Tsinghua Center for Life Sciences, Academy for Advanced Interdisciplinary Studies, Peking University, China

Metal-binding proteins (MBPs) constitute approximately one-third of the total protein repertoire and play crucial roles in diverse biological processes. The ensemble of MBPs in a specific living system is denoted as ‘metalloproteome’. Recently, we have developed a machine learning method named MetalNet [1] with superior performance for the prediction of metalloproteomes based on sequence coevolution. Nevertheless, there are still some limitations in MetalNet. First, the training set was limited to a relatively small structure dataset curated from PDB in 2016. Second, the computational efficiency of MSAs limited the ability of MetalNet to predict the metalloproteome of eukaryotic organisms. Third, the script-based application posed a certain level of technical barrier for wet-lab researchers to use MetalNet. Thanks to advancements in sequence search and clustering algorithms such as MMseqs2 [2], it has now become possible to obtain high-quality MSAs within an acceptable time frame. Furthermore, the rapid progress of protein language models (PLMs) has provided a more powerful tool for token representation, which has inspired us to further enhance the encoding methodology of MetalNet.

In this work, we developed MetalNet2 as a new generation of computational tool with enhanced prediction power for exploring metalloproteomes. The overall framework of MetalNet2 is illustrated in Fig. 1a. With an eight times larger dataset collected from the entire PDB (as of May 2023, Fig. 1b)and the introduction of ESM2 [3] pair encoding (Supplementary Method and Tables S1–2), the new prediction model outperformed MetalNet with an F1-score of 0.76, recall of 0.70 and precision of 0.83 on the holdout test set (Fig. 1c). Notably, the new model exhibited enhanced accuracy in predicting metal-binding pairs that are composed of two cysteine residues (Fig. 1d). This is because pairs of cysteines in disulfide bonds share a similar frequency matrix encoding (i.e. mutational behavior) with pairs of metal-chelating cysteines (e.g. involved in iron-sulfur cluster binding) in the old model, which tended to predict disulfide cysteines incorrectly as metal-binding residues. By leveraging encoding from ESM2, the new model is capable of capturing the global correlations among residues in the sequence, thereby allowing better discrimination of disulfide cysteines from metal-binding ones. Furthermore, we found that the new model had the ability to predict certain metal-binding sites located at the interface of homo-oligomers (Fig. S2). This case suggests that coevolution signals may occur between distant residue pairs [4] and MetalNet2 is able to capture such information without modeling the structure explicitly.

**Figure 1.**
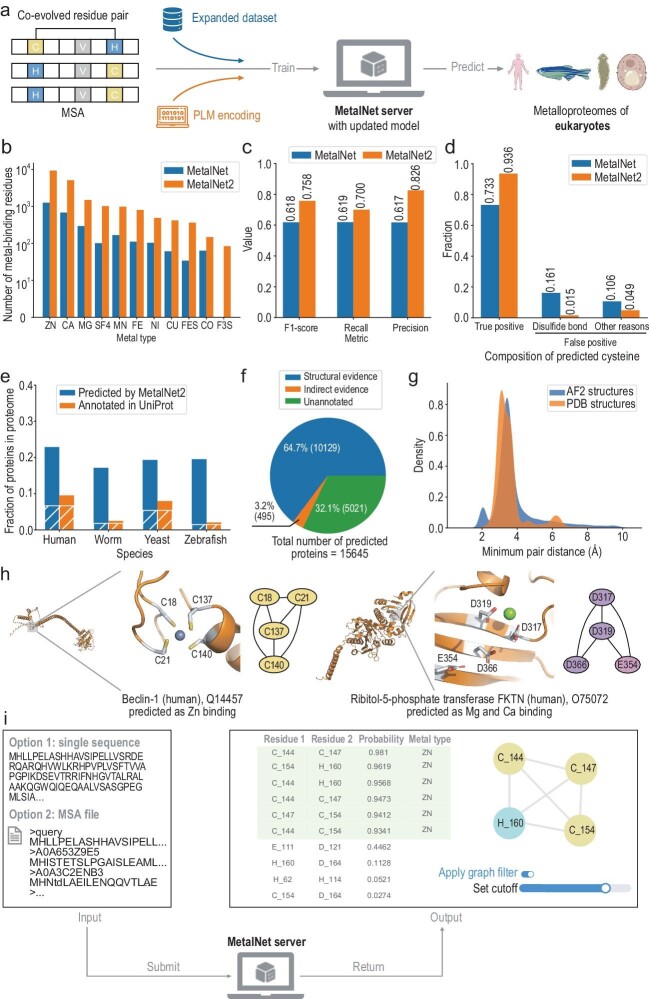
Development of MetalNet2 for predicting eukaryotic metalloproteomes. (a) Scheme of MetalNet2 and its application on the prediction of metalloproteomes of eukaryotes. (b) Distribution of metal types in the new MBPs dataset curated from PDB. (c) Overall comparison of the model performance on the test dataset between MetalNet vs MetalNet2. (d) Comparison of the model performance on predicting cysteine pairs between MetalNet vs MetalNet2 on the test dataset. (e) The percentage of predicted metal-binding proteins by MetalNet2 (blue) and annotated proteins in UniProt (orange) within each proteome. The overlapping proteins are highlighted in the diagonal stripe pattern. (f) Part of MetalNet2 predictions can be supported by structural evidence or indirect evidence from UniProt annotations. (g) Minimum pair distance distribution of predicted metal-binding residues by MetalNet2 according to the AlphaFold2 structure database (blue) and that of the metal-binding residues observed in PDB structure from the MetalNet2 dataset (orange). (h) Representative examples of predicted metal-binding proteins in the human proteome without previously known metal-binding annotations from UniProt. The left one is Beclin-1 (UniProt code: Q14457) with a predicted Zn binding site. The right one is FKTN (UniProt code: O75072) with a predicted Mg or Ca binding site. Both structures are modeled by the recently released AlphaFold3 server with protein sequence and a single metal ion as input. (i) An online server for MetalNet2.

As the aforementioned model does not provide the types of metals bound to the predicted metal-binding residue pairs, which is crucial for understanding the functions of proteins, we trained a separate multi-class classifier model using the types of metals bound in our curateddataset as labels (Fig. 1b) while keeping the same ESM2 encoding. Residue pairs predicted with potential metal-binding capabilities will undergo further prediction to determine specific metal types (Fig. S1). Compared to the motif-based matching strategy described in the original MetalNet, the new learning-based model was able to unambiguously make the prediction at the level of residue pairs instead of residue network topology. When it was applied to the holdout test set, MetalNet2 reached an overall average F1-score of 0.52 for metal-type prediction (Table S3) and was especially good at the prediction of Fe_4_S_4_ (0.86), Fe_2_S_2_ (0.85) and Zn (0.74). In particular, we evaluated the new model on an independent dataset of iron-sulfur cluster binding proteins dataset as compiled by Bak and Weerapana in 2023 [5], the results of which showed a coverage of 92% at the protein level and 88% at the residue level (Fig. S3).

Since the original MetalNet was only applied for the prediction of metal-binding sites in four prokaryotic proteomes, we extended MetalNet2 to explore the metalloproteomes of four representative eukaryotic species including human (*Homo sapiens*, UP000005640), zebrafish (*Danio rerio*, UP000000437), worm (*Caenorhabditis elegans*, UP000001940) and yeast (*Saccharomyces cerevisiae*, UP000002311). We evaluated the MSA quality of these four eukaryotes in comparison with those of four prokaryotes used in the original MetalNet. The results indicated a larger proportion of MSAs in eukaryotes with a *N_f_* value cutoff of 16, which is sufficient for extracting coevolved pairs (Fig. S4). In total, MetalNet2 predicted 15 645 MBPs that cover on average ∼70% of the currently annotated MBPs in UniProt (Fig. 1e). Among them, 10 624 (68%) could be supported by direct structural or indirect annotation evidences, leaving the rest of 5 021 (32%) as the resource of potentially novel MBPs for further characterization (Fig. 1f).

To evaluate the reliability of MetalNet2 predictions, we compared the distribution of minimal pair distances of the predicted metal-binding residues based on either the experimentally determined PDB structures or the computationally predicted AlphaFold2 models [6] (Fig. 1g). In general, the two distributions agree well with each other with the major peaks centering around 3 Å, suggesting a suitable coordination environment for accommodating single or multiple metal ions in the MetalNet2 predictions. Notably, the presence of a small peak around 2 Å in the distribution calculated based on the AlphaFold2 structures is attributed to the modelled disulfide bonds, which was predicted as metal-binding cysteine pairs. Considering the current version of AlphaFold2 does not explicitly take metal ions into account during the process of model generation and there have been reports of incorrectly modelled disulfide bonds in metalloproteins using AlphaFold2 [7], we envision that the ability of MetalNet2 in discriminating disulfides from metal-binding cysteine pairs holds the potential to contribute to a more accurate structural modeling of metalloproteins. Additionally, we highlighted two important proteins in the human proteome without previously known metal-binding annotations from UniProt (Fig. 1h), including the Beclin-1 (UniProt ID: Q14457), which plays a critical role in autophagy, predicted as Zn bound and the ribitol-5-phosphate transferase FKTN (UniProt ID: O75072), which is closely associated with cardiomyopathies, predicted as Mg or Ca bound. We utilized the recently developed AlphaFold3 model [8] to predict complex structures containing a single zinc ion or magnesium ion of the above two proteins. In both cases, as well as some other predicted metal-binding proteins by MetalNet2 (Fig. S5), the AlphaFold3 models supported a suitable geometric shape for local metal coordination. Since AlphaFold3 is currently only available as a web server with limited usage, we anticipate that a future open-source version of AlphaFold3 will fully unleash the power of MetalNet2 to provide a powerful tool for uncovering metal-binding proteins within the protein universe.

Finally, we developed an online server of MetalNet2 (https://www.chem.pku.edu.cn/wangchulab/metalnet) to facilitate public access and usage (Fig. 1i). The web server either takes an MSA file directly as input or searches the MSA using the Colabfold server [9] if a single protein sequence is provided. Usually after several minutes of calculation, the server will return a page demonstrating all the prediction results (Fig. 1i). The table on the left presents the probability of each coevolved CHED pair predicted with metal-binding activity. It is accompanied by a dynamically rendered graph illustrating the metal-binding network formed by these pairs. As reported in the original MetalNet, such a network cluster could increase prediction confidence and users are therefore given the option to flexibly adjust the cutoff to obtain results with different levels of stringency. Additionally, for each CHED pair that is predicted with high metal-binding probability by MetalNet2, the prediction of metal type will also be provided alongside. Last, we have uploaded to the server the predicted metalloproteomes of the aforementioned four eukaryotes (human, zerbrafish, worm and yeast) together with the updated prediction results of the four prokaryotic species (*Escherichia coli, Bacillus subtilis, Saccharolobus solfataricus* and *Halobacterium salinarum*) as reported in the original MetalNet.

In future, it might be possible to integrate the MetalNet2 workflow with AlphaFold to tailor structure prediction of metalloproteins. On a related note, incorporation of structural information from AlphaFold DB [10] as input for MetalNet2 might also help circumvent the dependence of MSA, which consumes a lot of computational resources with the growing sequence databases. Furthermore, despite the success of predicting iron-sulfur cluster or zinc-binding sites by MetalNet2, the accurate prediction of metal types is still challenging for Ca, Mg, Cu and other metals that occur in life with lower frequency. With the continuing improvement of protein structure prediction methods and the expansion of predicted protein structure databases, we envision that a structure-based approach will contribute significantly to the comprehensive annotation of metal-binding activities in both prokaryotic and eukaryotic proteomes. Collectively, we anticipate that MetalNet2 along with its public server will serve as an enabling tool for the community to expedite the exploration of metalloproteomes and their functions.

## Supplementary Material

nwae391_Supplemental_File

## CODE AVAILABILITY

Our code is available as open source at https://github.com/wangchulab/MetalNet2. The MetalNet2 server can be accessed at https://www.chem.pku.edu.cn/wangchulab/metalnet.
