# Supplementary material for: MetalNet2: an enhanced server for predicting metal-binding sites in proteomes
Source: Natl Sci Rev. 2024 Nov 5;11(12):nwae391. doi: 10.1093/nsr/nwae391 (PMC11660930; doi:10.1093/nsr/nwae391)
Supplement: nwae391_Supplemental_File [file nwae391_supplemental_file.docx]

Supplementary Materials for Manuscript

**MetalNet2: an enhanced server for predicting metal-binding sites in proteomes**

Feng Zhang^1,2^, Yao Cheng^1,2^, Boxin Xue^1^, Yiqin Gao^1,3,4^, Yuan Liu^1,2,*^, Chu Wang^1,2,5,*^

^1^Beijing National Laboratory for Molecular Sciences, College of Chemistry and Molecular Engineering, Peking University, Beijing, China.

^2^Synthetic and Functional Biomolecules Center, Key Laboratory of Bioorganic Chemistry and Molecular Engineering of Ministry of Education, Peking University, Beijing, China.

^3^Changping Laboratory, Beijing 102200, China.

^4^Institute of Systems and Physical Biology, Shenzhen Bay Laboratory, Shenzhen 518055, China.

^5^Peking-Tsinghua Center for Life Sciences, Academy for Advanced Interdisciplinary Studies, Peking University, Beijing, China.

This file contains:

**Supplementary Methods**

**Supplementary References**

**Supplementary Figures S1-S5**

**Supplementary Tables S1-S3**

**Supplementary Methods**

**Dataset collection**. Of ~200,000 protein structures in PDB that are deposited before May 2023, we defined the following filters using Biopython[1] to build a high-quality metal-binding dataset.

- The resolution of the protein structure should be less than 3 Å, which is a commonly used threshold for accurate metal-binding annotation[2].
- Metal in structure should be bound as a single metal ion, including Zn, Ca, Mg, Mn, Fe, Cu, Ni, Co; or three types of iron-sulfur cluster, including Fe_4_S_4_ (SF4), Fe_3_S_4_ (F3S) and Fe_2_S_2_ (FES). Na^+^ and K^+^ are not included as they are more often weakly bound to the surface of protein due to the crystallization conditions rather than biological functions[3].
- The number of residues that coordinate with the metal element using N, O, S in the backbone or side chain within 3 Å should be no less than 3. Site with other coordinated molecules except water (such as nucleotide, solvent molecule) is not allowed.

Redundant metal-chains were removed with a sequence identity threshold of 0.3 using MMseqs[4]. Then the dataset was split to the training set and the holdout test set with a ratio of 9:1 with no intersection of protein chains. Finally, only annotated CHED residues were retained as the model required.

**Model optimization**. Model hyperparameters, including coevolution pair extraction part and pair encoding part, were tuned in the training set by the five-fold cross-validation using AutoGluon[5]. Hyperparameters in pair extraction part included:

- Coevolution threshold. The value to define a coevolution pair for the output of MSA transformer[6]. Default: 0.1.
- MSA filter method and number of filtered sequences. The down sampling method for MSA as required by MSA transformer, including hhfilter[7] and max Hamming distance. Default: max Hamming to 64 sequences.
- MSA type. MSA type differs when it is searched against different database, including uniref MSA (uniref30_2103), envdb MSA (colabfold_envdb_202108) and merged MSA (merge the former two MSA). Default: uniref MSA.

Types of pair encoding included:

- Frequency matrix encoding as described in the original MetalNet model.
- Protein language model encoding. Specifically, the ESM2 residue encoding was used here, and three strategies were introduced to covert residue encoding to pair encoding: pairwise maximization, concatenation and average.

The model performance is evaluated as the F1-score in terms of all metal-binding residues. Hyperparameters in graph filter (as described in the original MetalNet model) with the best performance on validation set were chosen. The key parameters in TabularPredictor in AutoGluon is as follows:

TabularPredictor(
 label="label",
 problem_type="binary",
 eval_metric="f1",
).fit(
 train_data=train_data_df,
 tuning_data=tuning_data_df,
 presets='best_quality',
 use_bag_holdout=True,
)

A greedy search strategy was used to optimize the aforementioned hyperparameters. Specifically, hyperparameters in the pair encoding part were first optimized with that in the pair extraction part fixed as default. Then hyperparameters in the extraction part were tuned in the order presented above with the optimized hyperparameters in the encoding part.

**Model training for metal-type prediction**. Following the same procedure in the encoding tuning part, we additionally trained a multi-class classifier to predict metal-binding types of predicted pairs. It should be noted that, in the training stage, we only considered pairs that consist of two residues with the same metal-binding type; In the evaluation stage, if one residue was predicted with different metal-types in different predicted pairs, we still took this residue into account in the calculation of the related metrics in terms of those metal-types separately.

**Evaluation of MSA quality of eukaryotes**. We used MMseqs to search MSA of eukaryotes against the uniref30_2103_db database and MSAs of the four prokaryotes were from the paper of old MetalNet. Then we used gremlin (https://github.com/sokrypton/GREMLIN_CPP) to calculate Neff with sequence similarity threshold set as 0.8 and the normalized Neff (that is, Nf) was calculated by the definition from Zhang et al[8].

**Metal-binding annotation and evidence for four species**. We used the Swiss-Prot format file from UniProt to extract metal-binding annotations of proteomes in these four species. Specifically, if there is a 'BINDING' property with the record of anyone of the 11 metal types in the file, then it is regarded as the metal-binding annotated protein. And we used blast (https://blast.ncbi.nlm.nih.gov/doc/blast-help/downloadblastdata.html) to align the sequence in proteomes with that of metal-binding protein PDB structures, with e-value set to 1E-5. If there is intersection between the predicted residues and metal-binding residues in the alignment, then it is regarded as the protein with structural evidence.

**Supplementary References**

1. Cock PJA, Antao T, Chang JT *et al.* Biopython: freely available Python tools for computational molecular biology and bioinformatics. *Bioinformatics* 2009;**25**:1422–3.

2. Ye N, Zhou F, Liang X *et al.* A Comprehensive Review of Computation-Based Metal-Binding Prediction Approaches at the Residue Level. *Biomed Research International* 2022.

3. Babor M, Greenblatt HM, Edelman M *et al.* Flexibility of metal binding sites in proteins on a database scale. *Proteins: Structure, Function, and Bioinformatics* 2005;**59**:221–30.

4. Steinegger M, Soeding J. MMseqs2 enables sensitive protein sequence searching for the analysis of massive data sets. *Nature Biotechnology* 2017;**35**:1026–8.

5. Erickson N, Mueller J, Shirkov A *et al.* AutoGluon-Tabular: Robust and Accurate AutoML for Structured Data. *arXiv* 2020.

6. Rao R, Liu J, Verkuil R *et al.* MSA Transformer. *bioRxiv* 2021, DOI: 10.1101/2021.02.12.430858.

7. Steinegger M, Meier M, Mirdita M *et al.* HH-suite3 for fast remote homology detection and deep protein annotation. *BMC Bioinformatics* 2019;**20**.

8. Zhang C, Zheng W, Mortuza SM *et al.* DeepMSA: constructing deep multiple sequence alignment to improve contact prediction and fold-recognition for distant-homology proteins. *Bioinformatics* 2019;**36**:2105–12.

**Supplementary Figures**


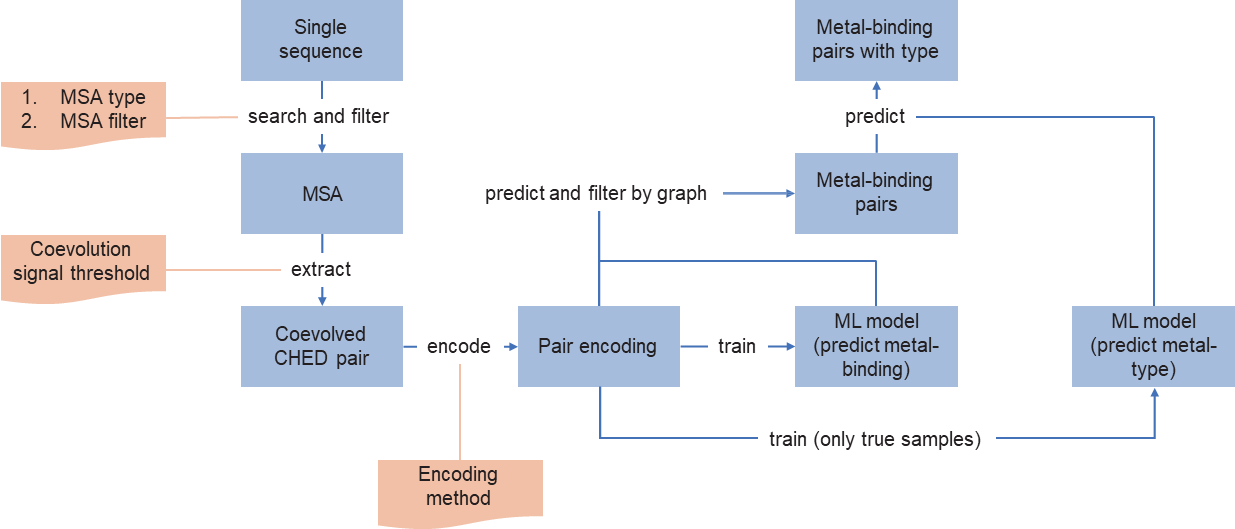


**Figure S1. Technical workflow of MetalNet2.** The overall workflow of model tuning (orange part), training and inference (blue part) is shown above. Of particular note is that the workflow involves two separate models for the prediction of metal-binding and the types of metals bound.


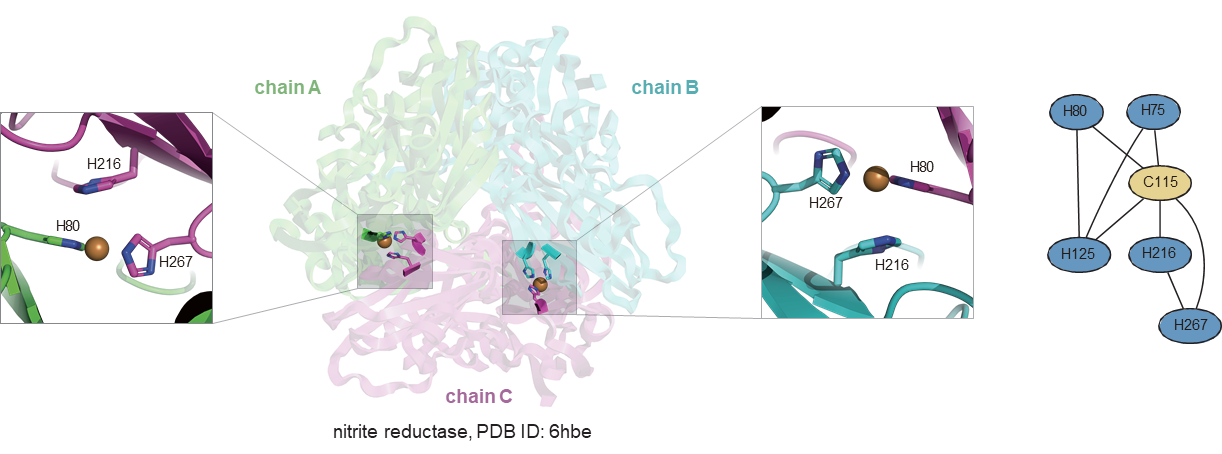


**Figure S2. Predicted metal-binding residues by MetalNet2 that are located at the interface** **of nitrite reductase** (PDB ID: 6hbe). MetalNet2 predicted a cluster assembled by predicted metal-binding pairs with a single sequence (chain C) as input. While H80 appeared distant from other predicted residues in the monomer structure suggesting a seemingly false positive prediction, it could be mapped to H80 in one monomer to form an inter-molecule metal-binding site with H216 and H267 in the other monomer according to the trimer structure.


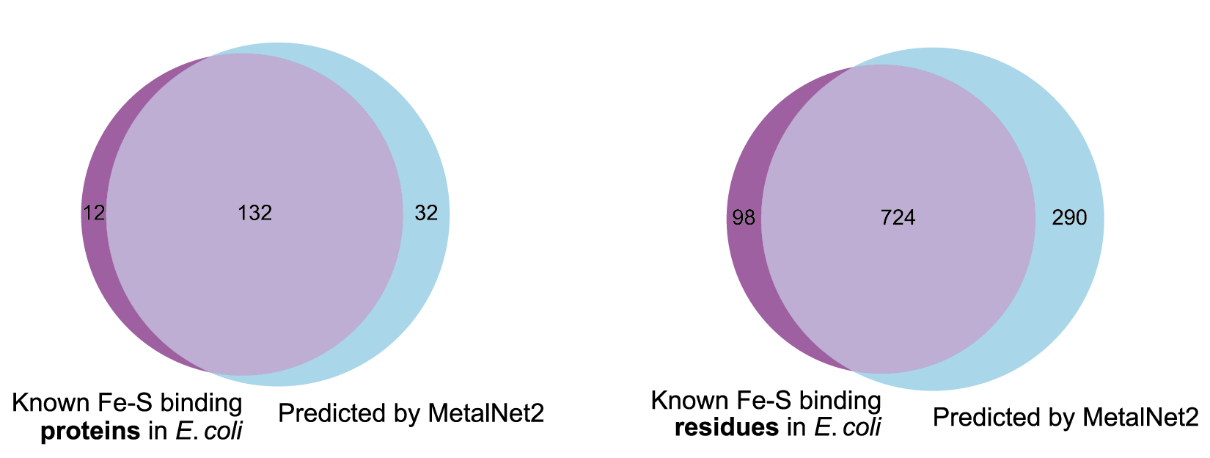


**Figure S3**. **Comparison of the predicted Fe-S binding proteins in *E.coli* by MetalNet2 with a known Fe-S binding protein dataset.** The dataset was compiled by *Bak et al*. The left Venn diagram presents the comparison result at the protein level while the right illustrates the comparison result at the residue level.


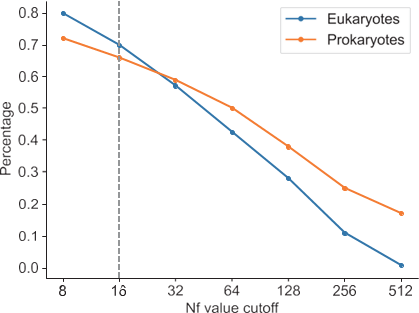


**Figure S4.** **The percentage of MSAs with different N_f_ value cutoff in eukaryotes and prokaryotes**. The N_f_ values of MSAs in four eukaryotic species and four prokaryotic species are calculated. The percentage in the figure is the proportion of MSAs with a higher N_f_ value than the cutoff value. Particularly, the grey dotted line indicates a larger proportion of MSAs in eukaryotes with a N_f_ value cutoff of 16 which is sufficient for extracting coevolved pairs.

**
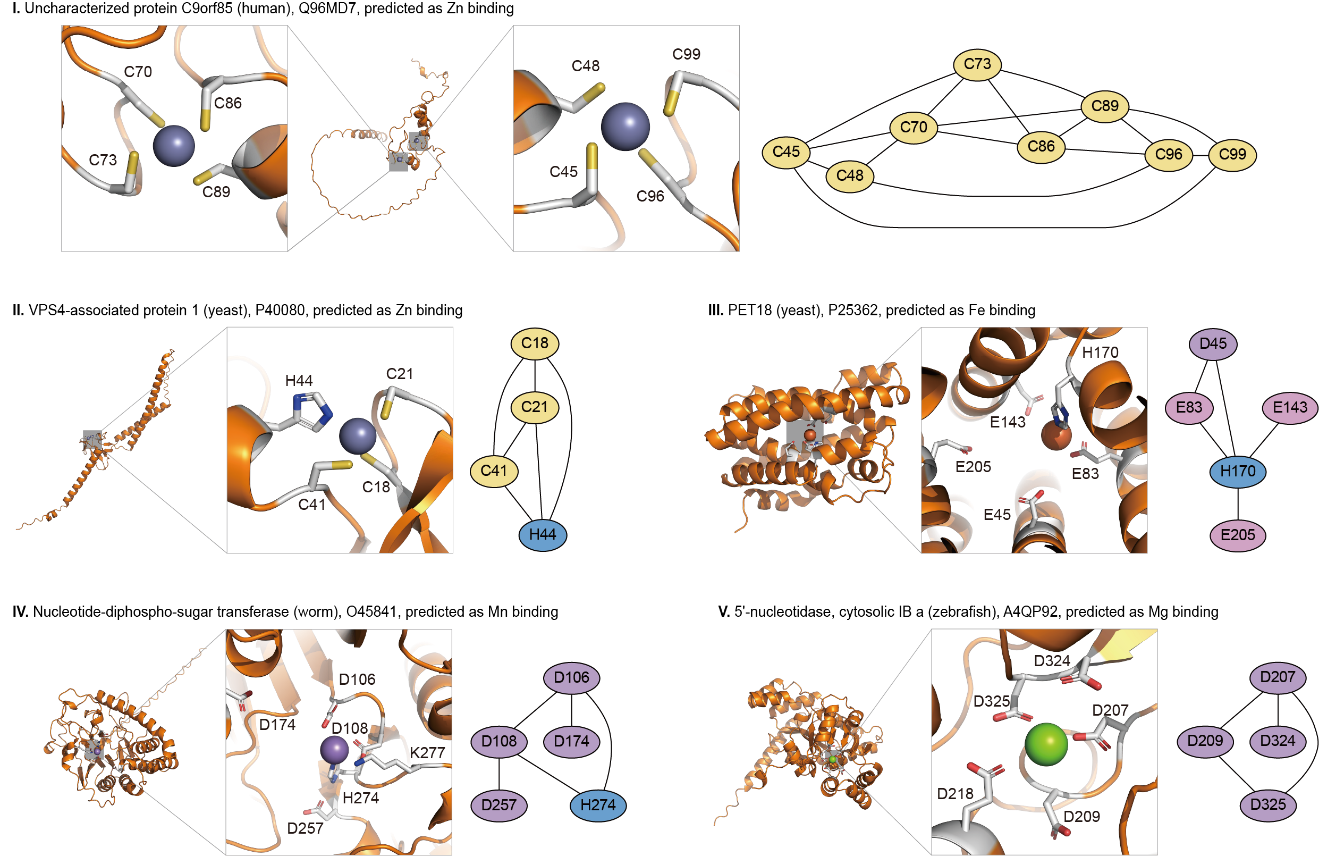
**

**Figure S5.** **Integrating MetalNet2 with AlphaFold3 to predict metalloprotein and model its metal-bound structure.** Five examples of metalloproteins predicted by MetalNet2 are shown. For each protein, the metal-binding network predicted by MetalNet2 was shown on the right and the AlphaFold3 models was shown on the left. The complex structure containing specific metal ion was modeled using the AlphaFold3 server with protein sequence and a single metal ion as input. For the protein Q96MD7, two copies of zinc ion were provided as MetalNet2 predicted two potential zinc-binding sites.

**Supplementary Tables**

**Table S1 Performance of the MetalNet2 model when the pair encoding part was tuned.** For comparison, the test result using the original MetalNet dataset is presented in the first line. For each condition, average F1-score and its standard deviation were shown.

| **encoding** | **strategy** | **F1_avg (%)** | **F1_std (10^-3^​)** |
| --- | --- | --- | --- |
| freq_mtx (old dataset) | - | 61.81 | - |
| freq_mtx | - | 66.55 | 2.905 |
| esm2 | max | 75.18 | 1.049 |
| esm2 | cat | 75.33 | 0.971 |
| **esm2** | **avg** | **75.37** | 1.226 |

**Table S2 Performance of the MetalNet2 model when the pair extraction part was tuned.** The empty cell indicates that the value is the same as the first row. For each condition, average F1-score and its standard deviation were shown.

| **coevo_threshold** | **msa_filter** | **num_seq** | **msa_type** | **F1_avg (%)** | **F1_std (10^-3^​)** |
| --- | --- | --- | --- | --- | --- |
| **0.1** | **MaxHamming** | **64** | **uniref MSA** | **75.37** | 1.226 |
| 0.01 |  |  |  | 73.19 | 3.578 |
| 0.2 |  |  |  | 74.37 | 1.241 |
|  | hhfilter |  |  | 73.46 | 0.528 |
|  |  | 16 |  | 74.79 | 1.147 |
|  |  | 32 |  | 75.21 | 1.801 |
|  |  | 128 |  | 74.74 | 2.392 |
|  |  |  | merged MSA | 74.98 | 1.932 |
|  |  |  | envdb MSA | 74.97 | 0.558 |

**Table S3** **Performance of the metal-type prediction model on the test dataset**.

| **metal type** | **precision (%)** | **recall (%)** | **F1-score (%)** |
| --- | --- | --- | --- |
| MN | 34.03 | 54.44 | 41.88 |
| F3S | 50.00 | 66.67 | 57.14 |
| CO | 35.71 | 25.00 | 29.41 |
| FES | 90.32 | 80.00 | 84.89 |
| CU | 64.29 | 64.29 | 64.29 |
| SF4 | 81.30 | 91.74 | 86.21 |
| FE | 22.45 | 41.51 | 29.14 |
| MG | 34.15 | 25.00 | 28.87 |
| NI | 16.67 | 12.82 | 14.49 |
| CA | 73.93 | 54.17 | 62.53 |
| ZN | 74.94 | 73.19 | 74.05 |
| macro average | 52.53 | 53.53 | 52.08 |
